# Prioritising meaningful outcomes in the practice evaluation of new chlamydia testing guidelines

**DOI:** 10.1016/j.lanepe.2025.101579

**Published:** 2026-01-08

**Authors:** Zoïe W. Alexiou, Nicole H.T.M. Dukers-Muijrers

**Affiliations:** a Department of Health Promotion, Care and Public Health Research Institute (CAPHRI), Maastricht University, the Netherlands; b Living Lab Public Health Mosa, Public Health Service South Limburg, the Netherlands

Werner and colleagues' timely work aimed to estimate the impact of the new Dutch guideline, which restricts *Chlamydia trachomatis* (CT) testing at Centers for Sexual Health to individuals with symptoms or partner notification. Using pre-guideline Amsterdam data, they estimated that over half of CT diagnoses, all asymptomatic, would remain undetected.^1^

Introduced in January 2025, the guideline is based on evidence that screening asymptomatic individuals does not significantly reduce CT prevalence or complications.^2^ Its goal is to de-implement and reduce unnecessary testing rather than maximise CT detection. Evaluation should therefore prioritise meaningful outcomes, such as antibiotic use and the incidence of symptomatic infections and complications. The counterfactual estimates presented assume continuation of historical trends and full guideline adherence, which is unlikely. While insightful, the data offer a preliminary estimate; nationwide real-world monitoring of relevant outcomes is essential and underway.

Symptom measurement is another consideration. Data were self-reported without specification of assessment methods. How symptoms are queried, through a general question or list, in person or on a form, affects reporting and testing decisions. Client awareness is crucial to prevent inequalities, as symptom perception is subjective and varies across populations.^3^ Additionally, prior evidence suggests that symptoms may carry differing risks of reproductive tract complications.^4^ Systematic recording is needed to ensure research quality and equitable access.

Finally, de-implementation is complex. Success depends on public communication, client behaviour, and especially professional practice and organizational environments.^5^ Monitoring and addressing determinants of clinical decision-making and the application of testing guidance are essential to assess real-world impact.

## Contributors

Conceptualization: ZWA.

Writing–original draft: ZWA, NHTMD-M.

Writing–review & editing: ZWA, NHTMD-M.

## Declaration of interests

The author declare that they have no conflicts of interest.
